# Survey of patient perceptions towards short-term mobile medical aid for those living in a medically underserved area of Swaziland

**DOI:** 10.1186/s12913-015-1186-4

**Published:** 2015-11-27

**Authors:** Yi-Hao Weng, Hung-Yi Chiou, Chi-Cheng Tu, Say-Tsung Liao, Patience Thulile Bhembe, Chun-Yuh Yang, Ya-Wen Chiu

**Affiliations:** Department of Pediatrics, Chang Gung Memorial Hospital, Chang Gung University College of Medicine, Taipei, Taiwan; School of Public Health, College of Public Health and Nutrition, Taipei Medical University, Taipei, Taiwan; Health Policy and Care Research Center, College of Public Health and Nutrition, Taipei Medical University, Taipei, Taiwan; Taiwan Medical Mission, Taipei Medical University, Taipei, Taiwan; Southern Africa Nazarene University, Manzini, Swaziland; Master Program in Global Health and Development, College of Public Health and Nutrition, Taipei Medical University, 250 Wu-Hsing Street, Taipei, 110 Taiwan; Department of Public Health, Kaohsiung Medical University, Kaohsiung, Taiwan

**Keywords:** Short-term medical mission (STMM), Humanitarian aid, Questionnaire, Patient, Satisfaction

## Abstract

**Background:**

An increasing number of short-term medical missions (STMMs) are being dispatched to provide humanitarian healthcare; however, extensive investigations on how recipient patients perceive STMMs are lacking. The current study evaluated the perceptions of patients toward medical services provided by a Taiwanese STMM in a resource-poor area of Swaziland.

**Methods:**

A structured questionnaire survey was completed by patients who had received medical services from the medical mission of Taipei Medical University in Swaziland in July 2014.

**Results:**

In total, 349 questionnaires were valid for the analysis. More respondents were female than male (69.6 % vs 30.4 %). The most common chief complaint was musculoskeletal problems (45.8 %), followed by respiratory symptoms (35.0 %). Most of the patients stated that their overall experience with the medical services was excellent (91.4 %). Universal patients would like to see the service provided in the future (99.7 %). Nearly 90 % of the patients were aware of how to take care of the medical problem they were diagnosed with. A majority of the patients comprehended what their medical providers said. Only a few patients did not understand what physicians said (5.2 %).

**Conclusion:**

Medical services provided by the STMM were helpful in resolving patients’ problems. The data have crucial implications for evaluating overseas mobile medical aid from the viewpoint of patients.

**Electronic supplementary material:**

The online version of this article (doi:10.1186/s12913-015-1186-4) contains supplementary material, which is available to authorized users.

## Background

Increasing numbers of health professionals are participating in humanitarian medical aid missions, which involve dispatching a group of experienced healthcare providers to resource-poor areas for volunteer service [1–5]. Such endeavors provide substantial benefits to the healthcare workers and to patients [6–8]. Medical aid missions are categorized into short- and long-term operations. There are three main categories of short-term medical mission (STMMs): emergency response, surgical, and mobile services [9]. Emergency-response services provide postdisaster medical relief worldwide; surgical services provide dental and surgical aid in regions where such services are generally unavailable [10]; and mobile services provide clinics in outlying villages where medical care is either lacking or inadequate [11].

Despite the benefits of STMMs, concerns have been raised regarding their long-term impacts on recipient communities [12–16]. Constraints have been cited, such as a lack of follow-up care, short duration, an excessive number of patients, language and cultural barriers, limited medications and supplies, and a lack of support services from local authorities [12, 14, 17]. Understanding beneficial partnerships between humanitarian organizations and host countries is imperative [18, 19]. A few studies have evaluated the perceptions of participating volunteer health workers [11, 20–22]. However, the perceptions of recipient patients have not been extensively investigated [23].

Several organizations in Taiwan have dispatched groups of health professionals, in the form of STMMs, to resource-poor countries. These efforts involve groups of healthcare personnel traveling to medically underserved areas far from Taiwan [20, 24, 25]. In these regions, obtaining medical services is difficult for most patients. Furthermore, there is asymmetric medical knowledge between patients and healthcare providers. In addition to medical conditions, past studies on medical aid missions have primarily focused on the behavior of health professionals [3, 7, 9, 20, 22, 26, 27]. Research investigating patients’ perceptions is rare [28]. In this article, we report the results of a questionnaire survey designed to explore the perceptions of patients toward mobile short-term assignments of volunteer medical services. We examined how medical aid missions were perceived in relation to experiences by patients in the Kingdom of Swaziland. This paper describes some implications of health evaluations for conducting successful STMMs.

## Methods

### Taiwan medical mission

A medical mission was dispatched to improve the healthcare environment of Swaziland by offering disease control education, training, guidance, experience, and methodologies as practiced in Taiwan. These efforts involved groups of healthcare personnel who traveled to medically underserved areas. Before the STMM medical service began, surveys were conducted to gather relevant information, including the required mission period, specialties of the mission personnel, locations of mobile clinics, licensure of medical care, and issues relating to the importation of drugs and equipment. Healthcare professionals for the STMM were recruited from various hospitals cooperating with Taipei Medical University and comprised a team of prominent specialist physicians, experienced nurses, pharmacists, and public health personnel. All members were fluent in English and had received disease prophylaxis, such as vaccinations, prior to departure [15].

The STMM covered all costs, including transportation and travel, daily expenses, logistical support, medications and supplies, laboratory capabilities, and accommodation. Medication formulary was established by the STMM. Drugs and equipment were prepared by participating hospitals in accordance with guidelines published by the World Health Organization and International Dispensary Association [27]. No medications were obtained locally. The medical services were designed to minimize any disruption to the culture of the host countries, conform to levels of local healthcare, and facilitate cooperation with local healthcare workers and nursing students.

The medical mission involved 110 personnel, namely 14 physicians, 18 nurses, 4 pharmacists, 13 public health managers, 11 drivers, and 50 volunteers and translators. Volunteers were responsible for language translation, triage, and measuring weight and height. Some Swazi personnel were recruited as translators to minimize the language barrier.

Some healthcare providers who participated in this STMM had been working for a long-term medical mission located at Mbabane Government Hospital, a national hospital in Mbabane, Swaziland. These healthcare providers were located at a driving distance of approximately 2.5 h from the STMM target location. The STMM physicians were internists, surgeons, dentists, pediatricians, obstetrician-gynecologists, and family physicians. Their subspecialties included allergy, rheumatology, pulmonology, neurology, neurosurgery, infectious disease, and radiology. In addition to medical care, the mission provided health education, including oral hygiene, wound care, physical massage, and disease prevention.

Radio advertisements and printed signs and fliers announced the forthcoming arrival of STMM in the target area. Swazi health officials also mobilized local health providers to spread the information about the STMM to patients in need.

### Study population

The location of this study was a resource-poor village in Endzingeni, Hhohho, Swaziland. All patients who received humanitarian medical services from the STMM in Swaziland during July 2014 were the survey targets. Children under 18 years of age were excluded.

### Survey administration

This study used a questionnaire survey. The questionnaire was anonymously distributed by volunteers to 499 patients after they received STMM medical services. The volunteers were composed of local residents who were highly aware of their language and culture. An orientation conference was conducted for volunteers before the questionnaire survey. In total, eight volunteers (three men and five women) assisted in collecting questionnaires.

### Questionnaire development

The development of an instrument for assessing patients was based on a comprehensive literature review of publications on existing questionnaires. The review led us to base our questionnaire on that of Maki et al. [18]. The questions for perceptions consisted of items for measuring the expectation, experience, preference, and adherence of patients toward medical services provided by the STMM. After the questionnaire was developed in English, the questions were translated into Swazi by a native speaker (see Additional file 1). Subsequently, back translation from Swazi into English was performed by another translator who was fluent in both Swazi and English to ensure the accuracy of translation.

### Validity and reliability

The questionnaire was examined by five experts to establish the content validity. Their expertise included global health, humanitarian aid (medical missions), survey methodology (statistics), international relations (diplomacy), and tropical medicine. Each expert had more than 20 years of work experience in their fields and fully understood the functions of the medical mission. The final questionnaire was modified according to the experts’ advice. In addition, the internal consistency of all indices was estimated using Cronbach’s alpha. In this survey, the content validity index of 0.82 and Cronbach’s alpha of 0.84 indicated sufficient validity and reliability, respectively, of the questionnaire parameters.

### Ethical considerations

The study protocol was approved by the Ethical Review Board of Taipei Medical University in Taiwan and the Scientific and Ethics Committee for Ministry of Health and Social Welfare Research in Swaziland. The questionnaire was accompanied by an introductory letter stating the purpose of the study and promising confidentiality. This letter was written in both English and Swazi. Patients willing to answer the questions and sign the consent form were enrolled in this study.

### Statistical analyses

All statistical analyses were conducted using SPSS 19.0 for Windows (SPSS, Chicago, IL, USA). Categorical variables were analyzed using the chi-squared test or Fisher’s exact test. For comparing the quantitative variables between the groups, the null hypothesis that there was no difference between each group was tested using analysis of variance. Significance was defined as *p* < 0.05.

## Results

The medical services were provided in camps built for the medical mission. On the day of the medical mission, patients lined up and were treated on a first-come, first-serve basis. At the end of the mission, no patient was dead or retained.

### Data collection

Questionnaires were distributed to patients who had received medical services provided by the STMM at Endzingeni. In total, 349 returned questionnaires with complete answers were valid for analysis (response rate: 69.9 %). Their demographic information is summarized in Table 1. More than half of the patients were aged > 60 years. There were more females than males. Most of the patients were married (90.5 %). Over half of the patients had no school education, and only 1.7 % of the patients had a college degree or higher. Furthermore, most of the patients were Swazis (99.7 %).

**Table 1:** Demographic data of 349 patients in Hhohho, Swaziland

Demographic information	Total ( %) *n* = 349
Age (years)	
20–40	28 (8.0)
41–50	39 (11.2)
51–60	78 (22.3)
> 60	204 (58.5)
Gender	
Female	243 (69.6)
Male	106 (30.4)
Marital status	
Single	29 (8.4)
Married	316 (90.5)
Other (divorced, widowed, etc.)	4 (1.1)
Education	
None	188 (53.9)
≤ 7 years	97 (27.8)
8–9 years	37 (10.6)
10–12 years	21 (6.0)
College	4 (1.1)
Masters or above	2 (0.6)
Ethnicity	
Swazi	348 (99.7)
Other	1 (0.3)

### Chief complaints of participating patients

The major problems of the patients were categorized into seven groups (Table 2): respiratory system (such as cough and rhinorrhea), cardiovascular system (such as hypertension), digestive system (such as diarrhea and vomiting), urogenital system (such as dysuria), musculoskeletal system (such as back pain), central nervous system (such as headache), and other problems (such as visual and skin problems). The most common problems were diseases of the musculoskeletal system (45.8 %), followed by respiratory system diseases (35 %). A total of 162 patients had more than one chief complaint (46.4 %). In addition, 92.7 % of the patients reported that they had access to medical providers who could follow up their medical problems.

**Table 2:** Chief complaints of the participating patients (*n* = 349)

Chief complaint	Number	Percent
Musculoskeletal system	160	45.8
Respiratory system	122	35.0
Urogenital system	13	3.7
Digestive system	36	10.3
Cardiovascular system	49	14.0
Nervous system	56	16.0
Other	113	32.4

### Characteristics of travel to receive medical services

**Fig. 1:**
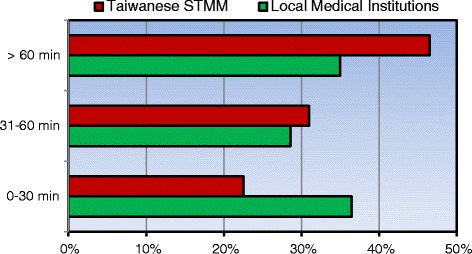
Travel times to the STMM and local medical institutions

The durations of time required to visit a local institution and the STMM were 75 and 91 min on average, respectively (Fig. 1). The traveling time to a local medical institution was shorter than that to the STMM (*p* < 0.001).

**Fig. 2:**
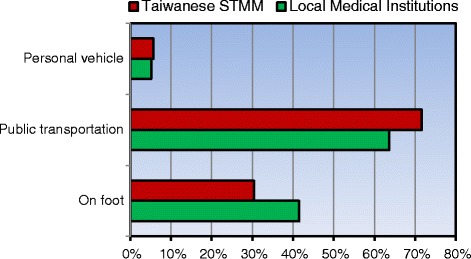
Mode of transportation to the STMM and local medical institutions

**Fig. 3:**
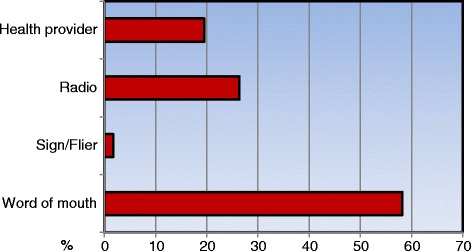
Information resources of medical services provided by the STMM

The methods of travel to the medical services are shown in Fig. 2. When traveling to a local institution, approximately two-thirds of patients used public transportation. There was a significant difference in the mode of travel used to receive medical services. Patients more often went to the STMM than to local medical institutions with public transportation (*p* < 0.001). Regarding how the patients knew about the STMM, most reported word of mouth as the information source, followed by the radio, a local health provider, and a sign or flier (Fig. 3).

### Perceptions of the STMM medical services

Most of the patients (91.4 %) rated the overall satisfaction with the medical services of the STMM as excellent (Table 3). In addition, most of the patients were able to take care of their medical problems (87.7 %). Furthermore, 99.7 % of the patients would have liked to see the STMM offer its services again. Nearly two-thirds of the patients (61.6 %) clearly understood what their medical providers said. Approximately one-third of the patients reported that they moderately understood what their medical providers said. Only a small proportion of the patients (5.2 %) did not understand what their medical providers said.

**Table 3:** Perceptions of medical care provided by the STMM

Perception	Total
	*n* = 349
Aware of what medical providers said	
Very much	215 (61.6)
So-so	116 (33.2)
Poorly	18 (5.2)
Able to take care of the medical problem	
Yes	306 (87.7)
No	14 (4.0)
Not sure	29 (8.3)
Overall satisfaction	
Poor	1 (0.3)
Average	29 (8.3)
Excellent	319 (91.4)
Expectation for STMM’s return	
None	1 (0.3)
Every year or more	16 (4.6)
Every 6 months	43 (12.3)
Every 4 months	66 (18.9)
Every 2 months or less	223 (63.9)

## Discussion

In this study, we evaluated the perceptions of patients treated by health professionals dispatched from Taiwan to Swaziland. These patients had few medical resources because of the remoteness of where they lived. Among approaches for evaluating STMMs, most researchers have focused on the quality of the host and visiting mission teams, examining parameters such as costs, efficiency, education, preparedness, and sustainability [11, 20–22, 29–32]. Studies investigating the impacts of STMMs on recipient patients are scant. Assessing patients’ needs is necessary because their satisfaction is a crucial outcome [18, 33–35]. To our knowledge, this is the first large-scale survey to evaluate the preferences and adherence of patients who received foreign humanitarian medical services.

Although medical aid for resource-poor countries has been provided for decades, the quality of medical care has not been adequately measured because of difficulties with evaluation models [18]. In this study, we conducted a questionnaire survey to investigate the healthcare quality of medical services provided by the STMM for people in Swaziland. Our study evaluated the expectation, experience, preference, and adherence of patients by asking matched questions. First, expectation was defined as whether the patients would like to see the service provided by the mission again. Second, experience was examined by asking the patients whether they understood what the medical providers said. Third, preference was described as whether patients were satisfied with the mission. Fourth, to assess adherence, we asked the patients whether they were able to take care of their diagnosed medical problem.

In this inquiry, we used the following questions to demonstrate that the STMM was helpful in resolving the patients’ problems. First, the overall experience with the medical services was positive. Up to 91.4 % of the patients expressed high satisfaction with the STMM’s medical services. Second, almost all the patients hoped to see the services provided in the future. These results indicate that the patients were willing to return for medical help. Third, approximately two-thirds of the patients comprehended the doctors’ explanations and understood how to treat their problems. These data suggest that although most of the patients encountered a language barrier with the STMM members, translation resolved this problem by enabling effective communication. Fourth, nearly all the patients could follow the doctors’ instructions. This is critical because the management of numerous diseases requires the cooperation of patients in their daily lives.

This survey was administered to 499 patients of one STMM. Some strategies were implemented to recruit this high number of patients. First, the STMM cooperated with local health providers to find patients in need. Second, the official health department provided a large area with an obvious target. This location enabled patients to conveniently access the STMM. Third, the STMM recruited local volunteers to assist in STMM management duties such as logistics and triage. These methods facilitated creating a support network assisting needy patients in visiting the STMM.

Our survey showed that approximately two-thirds of the patients received medical treatment at local institutions. We speculate that these patients came to the STMM for further management because they were not satisfied with their previous treatment at local institutions or because the STMM provided novel treatment. Overall, these patients were satisfied with the STMM services. Furthermore, the satisfaction of the patients who had received treatment in the past was higher than that of those without previous treatment (data not shown), suggesting that the medical services provided by the STMM seemed more satisfying than treatment by local institutions. In other words, STMMs can complement existing health services for patients in resource-poor settings. Although the travel time of patients to local medical institutions was shorter than that to the STMM, the participating patients still perceived high satisfaction with the STMM.

In this study, most of the patients learned of the STMM’s medical services through word of mouth. In addition, most of the patients came to the STMM on foot or by public transportation. Furthermore, many of the patients had multiple medical problems. Each patient spent an average of 1.5 h visiting the STMM. These data demonstrate that the resources in Swaziland – including medical supplies, transportation, and media – are deficient.

The lack of long-term follow-up has been criticized as a major problem with STMMs [18, 31]. In our study, approximately 93 % of patients claimed that medical providers were available for them to receive follow-up treatment. Nevertheless, almost all the patients wished to see the services provided by the STMM again in the near future. These data suggest that the healthcare quality of the STMM earned their trust. We posit several reasons for this trust. First, our STMM has a long-term partnership with official health organizations in Swaziland. Second, most of the healthcare providers in our STMM had been working in a local Swaziland hospital; thus, they knew the local culture well. Third, official native Swazi health workers participated in this STMM. These aspects of implementation enabled the STMM to reduce the cultural distance between the patients and providers.

The STMM has established a program for long-term follow-up. When patients were diagnosed with a disorder that required transfer, local health providers who worked in conjunction with the STMM assisted the patients in their referral to appropriate facilities. Because local health professionals are scarce in rural and remote areas, patients must travel great distances for medical care. In recent years, globalization has enabled health professionals to participate in humanitarian medical aid to overcome difficulties resulting from disparities in medical resources in underdeveloped countries [2, 30]. Our findings indicate that STMMs may serve as a means of providing medical help for patients in resource-poor areas.

There are some limitations in the interpretation of the study data. First, most of the patients were Swazis living in resource-poor areas. They may have had economic problems finding medical help. Thus, their high rating of satisfaction might simply be due to the STMM providing free medical services. Second, our questionnaire was a self-report instrument completed by patients and did not explore the quality of healthcare from the viewpoint of the health providers. Because this study was not an audit of actual practice, we cannot be sure that the self-reported changes actually reflected improved healthcare. Third, our questionnaire design was insufficient in providing a detailed representation of complex issues, such as transnational and transcultural contexts. A simple “yes or no” question cannot fully identify how patients perceived the STMM. Patients may answer “yes” to a question for a range of reasons. A questionnaire with open-ended questions is required to obtain comprehensive responses [28]. However, most people in Swaziland prefer to speak Swazi rather than English, making a qualitative method or interview difficult to conduct for foreign researchers. Recruiting more translators might facilitate overcoming the language barrier. Nevertheless, we do not believe that this questionnaire design limitation affected the validity or core findings of this study. Fourth, our questions mainly focused on current STMM service and did not consider long-term outcomes.

## Conclusion

In this study, we used a questionnaire survey to obtain information regarding patients’ views toward the provision of medical service by an STMM from Taiwan. This study represents the first large-scale analysis that addresses the perceptions of such patients. Our results demonstrate that most of the patients were satisfied with the medical services that they received and deeply welcomed the return of Taiwan Medical Mission. These findings suggest that STMMs are effective in providing healthcare in low-resource settings. We hope that this survey provides further impetus for integrating evidence of STMM effectiveness from the viewpoint of patients.

## Additional file

Additional file 1:
**Questionnaire in English and Swazi language.** (PDF 107 kb)

## Abbreviation

STMM: short-term medical mission

## References

[1] Rovers J, Andreski M, Gitua J, Bagayoko A, DeVore J (2014). Expanding the scope of medical mission volunteer groups to include a research component. Glob Health.

[2] Snyder J, Dharamsi S, Crooks VA (2011). Fly-By medical care: Conceptualizing the global and local social responsibilities of medical tourists and physician voluntourists. Glob Health.

[3] Mulvaney SW, McBeth J (2009). Medical humanitarian missions. Am Fam Physician.

[4] Panosian C, Coates TJ (2006). The new medical “missionaries”--grooming the next generation of global health workers. N Engl J Med.

[5] Walsh DS (2004). A framework for short-term humanitarian health care projects. Int Nurs Rev.

[6] Thompson MJ, Huntington MK, Hunt DD, Pinsky LE, Brodie JJ (2003). Educational effects of international health electives on U.S. and Canadian medical students and residents: a literature review. Acad Med.

[7] Martiniuk AL, Manouchehrian M, Negin JA, Zwi AB (2012). Brain Gains: a literature review of medical missions to low and middle-income countries. BMC Health Serv Res.

[8] Nelson BD, Lee AC, Newby PK, Chamberlin MR, Huang CC (2008). Global health training in pediatric residency programs. Pediatrics.

[9] Bajkiewicz C (2009). Evaluating short-term missions: how can we improve?. J Christ Nurs.

[10] Dupuis CC (2004). Humanitarian missions in the third world: a polite dissent. Plast Reconstr Surg.

[11] Chiu YW, Weng YH, Chen CF, Yang CY, Chiou HY, Lee ML (2012). A comparative study of Taiwan’s short-term medical missions to the South Pacific and Central America. BMC Int Health Hum Rights.

[12] Wolfberg AJ (2006). Volunteering overseas--lessons from surgical brigades. N Engl J Med.

[13] Bishop R, Litch JA (2000). Medical tourism can do harm. BMJ.

[14] Jesus JE (2010). Ethical challenges and considerations of short-term international medical initiatives: an excursion to Ghana as a case study. Ann Emerg Med.

[15] Solheim J (2010). Third world humanitarian medical trips--are they safe?. J Emerg Nurs.

[16] Hunt MR (2011). Establishing moral bearings: ethics and expatriate health care professionals in humanitarian work. Disasters.

[17] Hughes SA, Jandial R (2013). Ethical considerations in targeted paediatric neurosurgery missions. J Med Ethics.

[18] Maki J, Qualls M, White B, Kleefield S, Crone R (2008). Health impact assessment and short-term medical missions: a methods study to evaluate quality of care. BMC Health Serv Res.

[19] Suchdev P, Ahrens K, Click E, Macklin L, Evangelista D, Graham E (2007). A model for sustainable short-term international medical trips. Ambul Pediatr.

[20] Chiu YW, Weng YH, Chen CF, Yang CY, Lee ML (2014). Perceptions and efficiency of short-trm medical aid missions among key groups of health professionals. Eval Health Prof.

[21] Green T, Green H, Scandlyn J, Kestler A (2009). Perceptions of short-term medical volunteer work: a qualitative study in Guatemala. Glob Health.

[22] Bjerneld M, Lindmark G, Diskett P, Garrett MJ (2004). Perceptions of work in humanitarian assistance: interviews with returning Swedish health professionals. Disaster Manag Response.

[23] Sykes KJ (2014). Short-term medical service trips: a systematic review of the evidence. Am J Public Health.

[24] Chan G (1997). Taiwan as an emerging foreign aid donor: developments, problems, and prospects. Pac Aff.

[25] Tubilewicz C, Guilloux A (2011). Dose size matter? Foreign aid in Taiwan’s diplomatic strategy, 2000-8. Aust J Int Aff.

[26] Bussieres JF, St-Arnaud C, Schunck C, Lamarre D, Jouberton F (2000). The role of the pharmacist in humanitarian aid in Bosnia-Herzegovina: the experience of Pharmaciens Sans Frontieres. Ann Pharmacother.

[27] Johnson CA, Stieglitz N, Schroeder ME (2009). Opportunities and responsibilities for pharmacists on short-term medical mission teams. J Am Pharm Assoc.

[28] DeCamp M, Enumah S, O’Neill D, Sugarman J (2014). Perceptions of a short-term medical programme in the Dominican Republic: voices of care recipients. Glob Public Health.

[29] Naujokas A (2013). Raising the quality of care during medical missions: a survey to assess the need for clinical and anatomic pathology services in international medical missions. Arch Pathol Lab Med.

[30] Laleman G, Kegels G, Marchal B, Van der Roost D, Bogaert I, Van Damme W (2007). The contribution of international health volunteers to the health workforce in sub-Saharan Africa. Hum Resour Health.

[31] Levy ML, Duenas VJ, Hambrecht AC, Hahn EJ, Aryan HE, Jandial R (2012). Pediatric neurosurgery outreach: sustainability appraisal of a targeted teaching model in Kiev, Ukraine. J Surg Educ.

[32] Aziz SR, Ziccardi VB, Chuang SK (2012). Survey of residents who have participated in humanitarian medical missions. J Oral Maxillofac Surg.

[33] Kirsch TD, Perrin P, Burkle FM, Canny W, Purdin S, Lin W (2012). Requirements for independent community-based quality assessment and accountability practices in humanitarian assistance and disaster relief activities. Prehosp Disaster Med.

[34] Campbell SM, Roland MO, Buetow SA (2000). Defining quality of care. Soc Sci Med.

[35] Brook RH, McGlynn EA, Shekelle PG (2000). Defining and measuring quality of care: a perspective from US researchers. Int J Qual Health Care.

