# Supplementary material for: Survey of patient perceptions towards short-term mobile medical aid for those living in a medically underserved area of Swaziland
Source: BMC Health Serv Res. 2015 Nov 27;15:524. doi: 10.1186/s12913-015-1186-4 (PMC4661953; doi:10.1186/s12913-015-1186-4)
Supplement: Additional file 1: — Questionnaire in English and Swazi language. (PDF 107 kb) [file 12913_2015_1186_MOESM1_ESM.pdf]

No.：__________________________

Version 1.0/20130813

Questionnaire
(to be answered by **patients)**

In order to improve our quality of service, please kindly fill out the following questions.

1) What's your major problem today? __________________________________

2) Have you ever been treated for this problem before seeing this mission?
☐ a. Yes ☐ b. No

3) What kind of health information was provided to you today? *Multiple choices

☐ a. none ☐ b. medical care ☐ c. personal hygiene

☐ d. drug ☐ e. others, please specify

____

4) How well did you understand what the doctor(s) told you?

☐ a. very much ☐ b. so so ☐ c. poorly

5) Do you understand how to care for your health problem(s) in the future?

☐ a. Yes ☐ b. No ☐ c. not sure

6) How did you learn about this mission? *Multiple choices
☐ a. word of mouth ☐ b. sign/flier ☐ c. radio ☐ d. health provider

7) How long does it take you to get here from home?

About ______________hour(s),
by _____________ *Multiple choices
☐ a. on foot ☐ b. public transportation (ex: bus)
☐ c. personal vehicle (ex: car, motorcycle) ☐ d. others:________________ (ex: animal)

8) Please rate your overall experience with this mission.
☐ a. excellent ☐ b. average ☐ c. poor

9) How often would you like to see the services being provided by this mission?
☐ a. every 2 months ☐ b. every 4 months ☐ c. every 6 months
☐ d. every year ☐ e. never again

10) Will there be medical providers to follow up your problem?

☐ a. Yes ☐ b. No

11) How long does it take you to go to the **nearest** health provider (including clinic, health center, or hospital) from your home?

About ______________hour(s),
by _____________ *Multiple choices
☐ a. on foot ☐ b. public transportation (ex: bus)
☐ c. personal vehicle (ex: car, motorcycle) ☐ d. others:________________ (ex: animal)

No.：___________________________

Version 1.0/20130813

Personal information

12) Sex

☐ a. female ☐ b. male

13) Age

___________ years old

14) Current Marital status

☐ a. single

☐ b. married

☐ c. divorced

15) How many years of education do you have?

☐ a. never
☐ b. primary school
☐ c. secondary school
☐ d. high school
☐ e. college
☐ f. master or above

16) Ethnicity

☐ a. Swazis
☐ b. others__________

- interviewer’s signature：________________________________

Thank you very much for your time and cooperation!!
